# Attentional Harms and Digital Inequalities

**DOI:** 10.2196/30838

**Published:** 2022-02-11

**Authors:** Anna Hartford, Dan J Stein

**Affiliations:** 1 Brain-Behaviour Unit University of Cape Town Cape Town South Africa; 2 South African Medical Research Council Unit on Risk & Resilience in Mental Disorders Department of Psychiatry & Neuroscience Institute University of Cape Town Cape Town South Africa

**Keywords:** digital inequalities, attentional harms, excessive internet use, persuasive technologies, internet ethics, attention economies

## Abstract

Recent years have seen growing public concern about the effects of persuasive digital technologies on public mental health and well-being. As the draws on our attention reach such staggering scales and as our ability to focus our attention on our own considered ends erodes ever further, the need to understand and articulate what is at stake has become pressing. In this ethical viewpoint, we explore the concept of attentional harms and emphasize their potential seriousness. We further argue that the acknowledgment of these harms has relevance for evolving debates on digital inequalities. An underdiscussed aspect of web-based inequality concerns the persuasions, and even the manipulations, that help to generate sustained attentional loss. These inequalities are poised to grow, and as they do, so will concerns about justice with regard to the psychological and self-regulatory burdens of web-based participation for different internet users. In line with calls for multidimensional approaches to digital inequalities, it is important to recognize these potential harms as well as to empower internet users against them even while expanding high-quality access.

## Introduction

Over the past 3 decades, as the internet has become an increasingly indispensable tool for economic, social, and political inclusion, there has been extensive and warranted concern about the sorts of social inequalities, or stratifications, that are generated or exacerbated by digital exclusion.

Initially, first-level research on these digital divides focused predominantly on the issue of access or nonaccess to the internet. However, as access has widened and as the field has developed, it has become clear that more complex and multidimensional analyses of digital inequalities are necessary. In turn, focus has shifted from access alone to include the quality of access; questions regarding digital literacy and proficiency (referred to as *second-level digital divides*) [1]; and, more recently, the ability to generate concrete social and economic benefits via the internet (referred to as *third-level digital divides*) [2,3].

Increasingly, there has been growing concern about the prejudicial nature of the *harms*, and not only the benefits, that can accompany web-based participation, particularly with regard to surveillance, algorithmic bias, and predatory marketing [4-14]. Among the potential harms associated with digital access, which can be exacerbated by socioeconomic forces and factors, are what we will be calling *attentional harms*. The present nature of the internet has made it an extraordinary draw on human attention, which has been amplified by the internet’s design and commodification. The growing awareness of design features that encourage excessive use has necessitated an ethical reckoning regarding what is at stake when we introduce powerful forces of distraction into our lives at such an enormous scale or when human attention is treated as a commodity by the world’s most powerful corporations.

The acknowledgment of these factors has serious implications within multidimensional analyses of digital divides, especially insofar as our goal ought to be to provide digital access that empowers individuals and enriches their lives. In countries that have addressed earlier digital divides, the issue of attentional harms has become more pressing, and in some tech-heavy societies, being empowered to disconnect or having the option of “tech-lite” environments is increasingly seen as a privilege [15].

In many other countries, particularly those in the Global South, the issue of digital divides is understudied and underresearched, with the focus remaining predominantly on enduring first-level divides [16]. However, countries that are still addressing earlier digital divides now have the opportunity to do so in a more complex and multidimensional way, including by recognizing the potential harms and agential costs that can be generated by internet use, particularly when we are not adequately informed about and empowered against them.

## Attention Economies

Broadly speaking, persuasive design is the process of creating technologies in order to generate behavioral change. One can distinguish between the intended and unintended effects of persuasive design [17] and between persuasive design and outright manipulative, deceptive, or coercive design [18,19], though in some cases these distinctions will be vague. Fogg [20] famously founded the Stanford Persuasive Technology Lab (now the Behavior Design Lab) and developed a behavior model for persuasive design. Concern about the ethics of persuasive design has accompanied the field from the outset. Fogg [21] himself has been careful to say that these techniques should not be used for ignoble purposes and has proposed that education is key to empowering individuals against nefarious forms of persuasive design. Recent ethical debates concerning design principles have emphasized the roles of participatory design and design justice as ways of overcoming marginalization and oppression [6,22-25] as well as advancing design that is aligned with, rather than opposed to, human well-being and flourishing (a point to which we will soon return) [26].

The so-called *attention economy* has come to dominate the provision of many web-based services. As such, success and profitability often relies on maximizing user engagement; the more often and the longer users engage with products, the more data manufacturers are able to collect on them and the longer manufacturers have them as an audience for advertisers. In turn, the goal of many software developers has been to design products that generate habitual engagement and maximize use, drawing on techniques from applied psychology, neuroscience, and behavioral economics in their efforts.

Certain pervasive design features, such as “like” buttons (or their equivalents), push notifications, streaks, auto-play, and infinite scroll, have been especially successful in this regard and have proliferated across platforms. The success of some of these features in terms of maximizing use have often been attributed to intermittent variable rewards, which have long been linked to addictive behavior and are also associated with the addictive quality of slot machines [27,28]. At the level of our neural reward system, an uncertain reward generates a more significant dopamine response than those generated by a reliable reward. On prominent internet platforms, sophisticated machine learning technologies now endeavor to randomize rewards for each user. Insofar as these content “rewards” involve high arousal and extreme or divisive material, there are related concerns about social harms [29,30].

The effect of these design features on our behavior, and their draw on our attention, is patent. The habitual checking of certain internet platforms and incessant engagement with smartphones have become facts of life in many parts of the world. One report indicates that the average smartphone user checks their device over 70 times per day and swipes and interacts with it thousands of times [31]. Efforts to limit or reduce the time spent on devices, even among “ordinary” users, can require significant self-control and often results in failure [32]. The difficulty of focusing one’s attention while having ready access to the internet has also become renowned, with many people investing in internet and site-blocking software in order to aid concentration. The mere presence of a smartphone has been reported to adversely affect working memory and functional fluid intelligence [33].

Persuasive design focused on maximizing use has come under increased scrutiny in recent years. Public interest about the effects of persuasive technologies on our behavior, mental health, and well-being has grown significantly, informed by a public conversation featuring tech insiders (notably the former Google design ethicist Tristan Harris), policy makers, health specialists, and educators, among others [34]. Some have argued that the increasing sophistication of persuasive digital technologies and their personalized nature makes them a far deeper and more considerable threat to autonomy than more long-standing and familiar forms of persuasive design [35].

## Attentional Harms

The value of our attention, that is, our ability to direct our attention in meaningful ways and our capacity for sustained attention, is as yet underexplored and undertheorized territory [15,35]. However, as the draws on our attention and the power of digital distractions reach such staggering scales and as our ability to focus our attention on our own considered ends erodes ever further, the need to understand and articulate what is at stake has become pressing. Yet, the necessary ethical frameworks (and even vocabularies) for understanding the significance of these forces are presently underdeveloped. Williams [35] writes:

To date, the problems of “distraction” have been minimized as minor annoyances. Yet the competition for attention and the “persuasion” of users ultimately amounts to a project of the manipulation of the will. We currently lack a language for talking about, and thereby recognizing, the full depth of these problems. At individual levels, these problems threaten to frustrate one’s authorship of one’s own life.

Amplifying this sentiment, Dennett [36] has said that “this is perhaps the greatest risk to human political freedom that we’ve ever seen,” and that “an agent who controls your attention controls you.”

It is clear that what we pay attention to is closely related to our conscious awareness and, therefore, to deep aspects of our individual identity and what constitutes our experience of life. How we direct our attention has both voluntary and involuntary aspects, and our attention can be drawn and held in ways that are by no means indicative of our assessments of worth, including via persuasion, manipulation, and coercion. Insofar as we are compelled to direct ever more significant amounts of our attention in ways that we do not relate to on a deeper level or in ways that we would not reflectively endorse, there are elements of our very selfhoods that are at stake.

An emerging philosophical conversation has sought to situate attentional harms within existing paradigms for understanding what is necessary for human well-being. For the purposes of this viewpoint, we only summarize some of the approaches that have been taken and gesture to other moral frameworks that might be applicable to the issue of attentional harms. As seen above, in exploring the harms implicit in pervasive attentional loss, some philosophers have focused on personal autonomy [35,36]. Other philosophers, drawing on Wolf [37], have emphasized the importance of the construction of worth and meaning to human well-being and have argued that persistent distraction undermines the pursuit of this goal [38]. Still others have drawn on Nussbaum’s [39] capabilities approach, which asserts the moral importance of the freedom to achieve well-being and understands well-being in terms of an individual’s capabilities, to argue that the harms of excessive time spent on the internet can undermine the capabilities central to human dignity [40].

An enduring difficulty within this debate concerns how to distinguish between beneficial and harmful co-options of our attention. After all, sometimes our attention is drawn precisely because we are highly invested in something and consider it enduringly worthwhile and meaningful. This can also be true of the time we spend on the internet; there is no end to what can be pursued on the internet, including essential and otherwise worthwhile undertakings, and therefore no straightforward relationship between the time spent on the internet and the sorts of attentional harms with which we are concerned. Further, within certain parameters, escapism itself can be stress relieving and curative. Given this: how can we recognize the harmless aspects, and even the beneficial aspects, of attentional loss while simultaneously recognizing where our attention is being co-opted in harmful ways and where we are giving over large swathes of our lives and receiving little in return but angst, regret, and alienation from our web-based pursuits?

A deontological framework is potentially useful here; we can distinguish between cases where our attention is being used as a mere means to the ends of others and cases where it is being treated as an end in itself. Per this framework, in cases where our attention is used as a mere means, the sustained co-option of our attention can be construed as harmful. In cases where our attention is also treated as an end in itself—cases where our engagement is ultimately something we would reflectively endorse—the sustained co-option of our attention need not be harmful and may even be beneficial.

Another important consideration in appraising these attentional harms is what has been called the *indispensability thesis* [15]. The all-purpose nature of digital devices, which include a range of essential and work-related functions, has resulted in the use of such devices increasingly becoming a requirement in both our personal and professional lives (and never more so than during the COVID-19 pandemic). This increasing indispensability has long motivated concern about digital inequalities in terms of the quality of access. However, the fact of indispensability also raises different ethical questions, and there is a sense in which the indispensability of (near-constant) internet access undermines full-fledged consent to the risks and deleterious effects associated with such access and engagement, given that there is increasingly no realistic alternative [15].

The current nature of the internet exacerbates this difficulty. In most cases, people cannot keep only the “essential” internet on them (ie, the parts they need to function or pursue their goals); people must always have access to the whole thing, including parts that might be sources of compulsion and regret. As Hanin [15] puts it:

Whereas no sane adult must smoke, use drugs, consume sugary foods, or gamble as a precondition to leading a fulfilling life or excelling in a profession, many sane adults have no practical way of avoiding often prolonged entanglement with digital ecosystems in the workplace and their personal lives. This entanglement poses formidable psychological challenges for self-regulation.

We agree with scholars who argue that widespread and systemic attentional harms should not be downplayed or dismissed and that they can potentially be deeply undermining to human agency and well-being. Respect for individuals ought to include respect for their attention, and any aspirational notion of the value of human life and the conditions for human flourishing ought to recognize the importance of our engaged presence and the ideal of achieving a sense of meaning and worth within our lives [26,41]. Insofar as digital distractions stymie these pursuits—often intentionally and on an utterly unprecedented scale—we are warranted in resisting or seeking to alter the forms these technologies take within our lives.

## Attentional Inequalities

As research into digital inequalities has emphasized, in expanding access to web-based services, it is crucial to acknowledge that not all internet access is equal. This inequality also pertains to attentional harms. The potential burdens of internet access, including the psychological and self-regulatory burdens that we have been describing, are very differently felt by different users depending on their device, their digital literacy, their awareness of these potential harms, and their recourse to strategies for avoiding them.

In societies where inequalities related to internet access have largely been overcome, socioeconomic vulnerability sometimes correlates with more time, rather than less time, on certain platforms, including social media and digital games [8,42]. In some places, “tech-lite” environments are becoming the ultimate privilege. For example, it is a much-reported fact that many Silicon Valley insiders send their children to the deliberately “tech-lite” Waldorf School [43]. Advocating for government-provided “tech-lite” environments, Hanin (writing from the United States of America) emphasizes that “poor and rich alike should have access to such settings, which may otherwise risk becoming a luxury for the few” [15].

Societies that are addressing earlier digital divides, including those in the Global South, should do so with cognizance of the emerging complexities and new stratifications regarding the potential attentional costs and burdens of access. In other words, the most empowering forms of connectivity are those that also empower us with strategies and effective methods for disengaging and disconnecting when we recognize, and are enabled to recognize [44], that time spent on the internet is impeding our own considered ends rather than serving them.

Research has also suggested that socioeconomic vulnerability is a risk factor for developing clinically significant internet addiction [45]. Our earlier points about indispensability are relevant here too. One important consideration is determining how feasible it is for individuals to control their access, especially among those who are vulnerable to internet addiction or problematic use. As Christakis [46] writes:

Cultural influences both mandate and facilitate that we spend time “online,” meaning that teetotalism is not an option. Given our current understanding that there is a genetic predisposition to behavioral addictions, we may be going a long way toward ensuring that the entire susceptible population develops them.

There are a range of straightforward socioeconomic factors that potentially contribute to attentional harms. Low-income internet users are much more likely to go on the internet by using their phone rather than a computer [8,47], which increases data vulnerability and, in many cases, limits control. Moreover, the ability to afford a more expensive smartphone provides a far higher degree of data privacy and greater control over which apps and services can be removed from a device or controlled within it, including those that someone might find to be compulsive or habit forming. A person who can afford to buy an iPhone (Apple Inc) or a parent who can afford to buy one for their child therefore has a far greater range of control over the time spent on their device and other use limits compared to that of someone who cannot afford to buy one [34]. Android phones (which cost one-third of the price of iPhones) have also been reported to collect significantly more personal data [48].

Data vulnerability has primarily been assessed through the lens of privacy and security [8,11]. However, another aspect of this vulnerability relates to attentional harms and the efficacy of targeted content. Increased data collection can generate a vicious cycle in which data can be deployed to better maximize use (through artificial intelligence–driven personalized recommendations and randomized rewards), which in turn allows more data to be gathered [8,15]. Relatedly, these factors increase vulnerability to predatory marketing tactics [13,49].

Socioeconomic inequalities in the burdens of attentional harms are liable to increase in coming years as divisions concerning who is aware of these harms emerge and as wealthier internet users buy their way out of some of the more noxious aspects of the web-based attention economy [44]. In considering ways to move away from the attention economy model, the most obvious suggestion is to require users to pay for services. In recommending regulations on persuasive technologies, Williams [35] suggests that “companies could be expected (or compelled, if necessary) to give users a choice about how to ‘pay’ for content online – that is, with their money or with their attention.” Aspects of this choice are already prevalent on the internet, with the distinction between free and premium services. As Roose writes [50]:

Today’s internet is full of premium subscriptions, walled gardens and virtual VIP rooms, all of which promise a cleaner, more pleasant experience than their free counterparts.

At present, available methods for mitigating attentional harms require significant digital literacy. In the first place, one must recognize such forces and the designs that exacerbate them, and one must further recognize the available means for resisting them. One study, which used education as a proxy for socioeconomic status, found that while internet users with both low and high education levels recognize the attentional burdens of being on the internet, especially with regard to wasted time, highly educated users were much more likely to intervene or to consider that time spent on the internet is potentially detrimental to their self-actualization [51]. Provided that one is aware of these options and, in many cases, is also able to pay for them, one can partially mitigate digital distraction by investing in site-blocking software, ad-blocking software, apps that generate screen time alerts, or devices that disable one’s internet connection for certain hours. People can also hide more of their personal information, encrypt their browsing history, and invest in apps and plug-ins that overwrite certain persuasive design features and shift default settings to those that reduce use rather than amplify use [52].

If the goal of overcoming digital inequalities is to empower individuals and enhance their well-being and their access to opportunities, we have to be cognizant of aspects of digital access that are deliberately *disempowering*, undermine our pursuit of meaning and worth within our lives, and ultimately serve powerful corporate interests that might well conflict with the interests of individual users.

The fact that these burdens can be compounded by socioeconomic factors generates further moral reasons to support decent minimum standards for design regulation and user controls (regardless of the cost of a device and regardless of whether services are free or paid for), since these burdens raise issues of justice with regard to the distribution of these essential controls and, in turn, raise issues of justice with regard to who will be empowered to better safeguard their attention as an end in itself and whose attention will be treated as a mere means to the ends of others.
